# Predicting Mortality in Low-Income Country ICUs: The Rwanda Mortality Probability Model (R-MPM)

**DOI:** 10.1371/journal.pone.0155858

**Published:** 2016-05-19

**Authors:** Elisabeth D. Riviello, Willy Kiviri, Robert A. Fowler, Ariel Mueller, Victor Novack, Valerie M. Banner-Goodspeed, Julia L. Weinkauf, Daniel S. Talmor, Theogene Twagirumugabe

**Affiliations:** 1 Department of Medicine, University of Rwanda, College of Medicine and Health Sciences, Kigali, Rwanda; 2 Department of Medicine, Division of Pulmonary, Critical Care and Sleep Medicine, Beth Israel Deaconess Medical Center and Harvard Medical School, Boston, MA, United States of America; 3 Department of Anesthesia, University of Rwanda, College of Medicine and Health Sciences, Kigali, Rwanda; 4 Department of Critical Care Medicine and Department of Medicine, Sunnybrook Hospital, Interdepartmental Division of Critical Care, University of Toronto, Toronto, ON, Canada; 5 Department of Anesthesia, Critical Care and Pain Management, Beth Israel Deaconess Medical Center and Harvard Medical School, Boston, MA, United States of America; 6 Clinical Research Center, Soroka University Medical Center, Faculty of Health Sciences, Ben-Gurion University of the Negev, Beer Sheva, Israel; 7 Department of Anesthesia, University of Virginia, Charlottesville, VA, United States of America; Azienda Ospedaliero-Universitaria Careggi, ITALY

## Abstract

**Introduction:**

Intensive Care Unit (ICU) risk prediction models are used to compare outcomes for quality improvement initiatives, benchmarking, and research. While such models provide robust tools in high-income countries, an ICU risk prediction model has not been validated in a low-income country where ICU population characteristics are different from those in high-income countries, and where laboratory-based patient data are often unavailable. We sought to validate the Mortality Probability Admission Model, version III (MPM_0_-III) in two public ICUs in Rwanda and to develop a new Rwanda Mortality Probability Model (R-MPM) for use in low-income countries.

**Methods:**

We prospectively collected data on all adult patients admitted to Rwanda’s two public ICUs between August 19, 2013 and October 6, 2014. We described demographic and presenting characteristics and outcomes. We assessed the discrimination and calibration of the MPM_0_-III model. Using stepwise selection, we developed a new logistic model for risk prediction, the R-MPM, and used bootstrapping techniques to test for optimism in the model.

**Results:**

Among 427 consecutive adults, the median age was 34 (IQR 25–47) years and mortality was 48.7%. Mechanical ventilation was initiated for 85.3%, and 41.9% received vasopressors. The MPM_0_-III predicted mortality with area under the receiver operating characteristic curve of 0.72 and Hosmer-Lemeshow chi-square statistic p = 0.024. We developed a new model using five variables: age, suspected or confirmed infection within 24 hours of ICU admission, hypotension or shock as a reason for ICU admission, Glasgow Coma Scale score at ICU admission, and heart rate at ICU admission. Using these five variables, the R-MPM predicted outcomes with area under the ROC curve of 0.81 with 95% confidence interval of (0.77, 0.86), and Hosmer-Lemeshow chi-square statistic p = 0.154.

**Conclusions:**

The MPM_0_-III has modest ability to predict mortality in a population of Rwandan ICU patients. The R-MPM is an alternative risk prediction model with fewer variables and better predictive power. If validated in other critically ill patients in a broad range of settings, the model has the potential to improve the reliability of comparisons used for critical care research and quality improvement initiatives in low-income countries.

## Introduction

Intensive Care Unit (ICU) risk prediction models estimate expected hospital mortality based on patient characteristics. The models facilitate case-mix adjustment in research, across-institution benchmarking, and individual ICU quality improvement evaluations.[1] The first adult ICU risk prediction model to gain broad use, the Acute Physiology and Chronic Health Evaluation (APACHE) model, was developed in 1981 using a sample of 805 patients from two hospitals.[2] Since then, numerous other ICU models have been developed, validated, and modified to improve goodness-of-fit.[1, 3–14]

While the large majority of resource-intensive critical care occurs in middle- and high-income countries, critical care does occur in low-income countries, and critical illness disproportionately affects people in low-income countries.[15–17] Attempts to improve the research methods and quality of critical care in low-income countries are hindered by the dearth of context calibrated risk prediction models with feasible data collection requirements. Data collection burden is an important barrier to the use of current risk prediction models in low-income countries. Even in the United States, only 10–15% of ICUs regularly use predictive models for quality improvement, and the burden of data collection is an oft-cited reason.[18] In addition, the characteristics of ICU patients in low-income countries may be quite different from those in high-income countries,[19] thus raising the question of whether models developed from populations in high-income countries will be accurate for low-income countries.[7, 20] To our knowledge, the only ICU model developed in a low-income country is the “Clinical Sickness Score” (CSS), created by Watters et al in 1989 based on 624 ICU admissions to a university teaching hospital in Zambia.[21] This model did not use current statistical methods for assessing discrimination and calibration, and it was never validated in another population.

In order to facilitate ICU research and quality improvement efforts in low-income countries, a risk prediction model that is calibrated for the population and relies on a parsimonious, easily collected set of variables is needed. In our study, we first assess the performance of the Mortality Probability Admission Model, version III (MPM_0_-III) in patients admitted to two ICUs in Rwanda; this model was chosen because it is the only ICU risk prediction that has been validated in a large cohort and is not dependent on laboratory values. We then sought to develop a new model with better predictive ability in this population and with less data collection burden.

## Material and Methods

### Study oversight

The study was conducted at the University Teaching Hospital of Kigali and the University Teaching Hospital of Butare, both affiliated with the University of Rwanda. Ethical and scientific committees at the University of Rwanda approved the study, as did the Committee on Clinical Investigations at Beth Israel Deaconess Medical Center in Boston, USA. Requirement for individual patient-level consent was waived given the determination of minimal risk to patients.

### Study population and setting

Consecutive patients admitted to the ICU at the University Teaching Hospital of Kigali (6 ICU beds) and the ICU at the University Teaching Hospital of Butare (5 ICU beds) were enrolled from August 19, 2013 to October 6, 2014. These hospitals are both public referral academic teaching hospitals, and contain all public adult ICU beds in Rwanda. For purposes of this analysis, we excluded all patients younger than 15 years old.

### Definitions

Sepsis was defined using criteria outlined by the most recent international consensus groups at the time of the study: at least two of four Systemic Inflammatory Response Syndrome (SIRS) criteria and suspected infection.[22–24] Severe sepsis was defined as these criteria plus evidence of organ hypoperfusion or dysfunction. Septic shock was defined by the presence of all of these, with mean arterial pressure<60 mmHg or systolic blood pressure <90 mmHg despite adequate fluid resuscitation. The Acute Respiratory Distress Syndrome (ARDS) was defined based on the Berlin definition: bilateral opacities on chest radiograph consistent with edema; lack of evidence of left atrial hypertension; positive end expiratory pressure (PEEP) of at least 5 cm H_2_O, and occurrence within one week of a clinical insult.[25] Since arterial blood gases were not available throughout the duration of the study, we used a hypoxia cutoff of S_p_O_2_/F_i_O_2_, ≤ 315 (excluding any S_p_O_2_ >97%), based on a study that derived and validated the correlation between P_a_O_2_/F_i_O_2_ ≤ 300 and S_p_O_2_/F_i_O_2_, ≤ 315.[25, 26]

Operational definitions for the MPM_0_-III variables were based on those from the initial MPM_0_-III development and validation study.[6] MPM_0_-III consists of 16 variables assessed within one hour of ICU admission. As in the initial development and validation of MPM_0_-III, missing values were treated as normal. Discharge and death diagnoses were determined using information in the chart. Diagnoses were classified using the Agency for Healthcare Research and Quality’s (AHRQ) Clinical Classifications Software (CCS). This maps 17 broad diagnostic categories and a total of 260 specific diagnoses to ICD-10 codes.[27]

### Data collection and quality assurance

One physician and one nurse at each site prospectively recorded data during each ICU admission. Data were collected on paper forms, and then entered into a web-based Research Electronic Data Capture (REDCap) electronic data capture tool.[28] Data collected included demographic information, insurance status, hospital admission data, ICU admission data, MPM_0_-III variables (acute and chronic organ-specific conditions), laboratory values when available, variables to determine the presence of sepsis, severe sepsis, septic shock within 24 hours of ICU admission, and ARDS at any time during ICU admission, interventions performed during admission, discharge diagnoses, and in-hospital vital outcomes. Training in data collection, including detailed operational definitions for each data element, was provided to the data collectors prior to beginning data collection. Study investigators randomly selected and reviewed 3 charts each week for eight weeks (approximately 40% of all charts in those weeks) to assess for inter-rater agreement in interpretation of data elements. Throughout the study, data validation reports were produced to check data accuracy and to identify any areas of systematic error. Any inter-rater disagreement was corrected for the chart in question, and other prior charts with potential for similar disagreement were reviewed. Biweekly meetings addressed inconsistencies, and study investigators were available every day for data collector questions.

### Statistical Analysis

The primary outcome was in-hospital mortality. For descriptive variables, we calculated proportions for categorical variables and medians with interquartile ranges (IQRs) for continuous variables. We performed univariate analyses using Student’s t-tests and chi-square tests with significance set at p<0.05.

Multivariate logistic regression models were used for in-hospital mortality prediction. We chose variables from the univariate analyses, based on their predictive power (as determined by a p value< 0.05) as well as their ease of capture based on our experience, the proportion of missing values in our dataset, and their clinical significance. We tested each of the 16 independent variables in the MPM_0_-III model, as well as additional variables: HIV status (positive/negative), HIV treatment status (on/off antiretroviral medications), age, insurance status (national public, private, or none), time prior to receiving care (in days), admission and ICU vital signs (systolic and diastolic blood pressure, pulse, temperature, respiratory rate, oxygen saturation, Glasgow Coma Scale score), reasons for ICU admission, presence or absence of sepsis, severe sepsis, or septic shock, presence or absence of ARDS, and blood laboratory values (sodium, potassium, creatinine, urea, white blood cell count, hemoglobin, platelets, aspartate transaminase, and alanine transaminase).

The final parsimonious model became the Rwanda Mortality Probability Model (R-MPM). We used area under the receiver operating characteristic curve (AUC, or c-statistic) to assess model discrimination (how effectively a model assigns a higher probability of death to a non-survivor than a survivor).[3] Generally, an AUC of 0.70–0.80 is acceptable, 0.80–0.90 good, and greater than 0.90 excellent. We calculated a Hosmer-Lemeshow statistic and p-value to assess calibration (how well the model predicts outcomes across the entire spectrum of risk, assessing predicted versus actual outcomes in each decile of risk.)[6] Acceptable calibration is generally defined as a non-significant Hosmer-Lemeshow value (p>0.05). Since we did not have a separate validation cohort, we also performed internal validation with bootstrapping in order to estimate the optimism in our model, expressed as a confidence interval (CI) around the AUC. We compared the model performance to the MPM_0_-III. We completed all analyses using SAS software, version 9.3.

## Results

### Patient characteristics, interventions, and outcomes

There were 427 patients admitted to the ICUs during the study period; we were unable to locate discharge vital status on two patients after extensive searching, so we had outcomes data on 425 patients. Patient characteristics are presented in Table 1 and S1 Table. About half of patients were male, and median age was 34 (IQR 25–47) years. Patients were insured in 93.3% of cases, with the vast majority insured by the national community-based medical insurance.[29] Admissions largely originated from district hospital transfers [72.3%), with an additional 10.2% coming directly from an accident site. The median time spent sick at home before seeking healthcare was one day, and the median time spent at a district hospital prior to referral was one day.

**Table 1:** Patient characteristics at hospital admission.

	Number of Patients*	Full Cohort (n = 427)**	Survivors (n = 218)	Non-survivors (n = 207)	P Value
**Demographic characteristics, n (%)**					
Male	427	209 (49.0)	107 (49.1)	101 (48.8)	0.952
Age in years, median (IQR)	427	34 (25–47)	34 (25–45)	34 (26–49)	0.271
Insured	420	392 (93.3)	201 (93.1)	189 (93.6)	0.835
**Presenting characteristics, n (%)**					
Patient arrived from:	422				
Transfer from district hospital		305 (72.3)	146 (67.6)	157 (77.0)	0.043
Site of accident		43 (10.2)	22 (10.2)	21 (10.3)	0.986
Home		33 (7.8)	24 (11.1)	9 (4.4)	0.010
Transfer from another referral hospital		27 (6.4)	17 (7.9)	10 (4.9)	0.210
Transfer from private clinic		5 (1.2)	2 (0.9)	3 (1.5)	0.678
Referral from health center		2 (0.5)	0 (0)	2 (1.0)	0.237
Other		7 (1.7)	5 (2.3)	2 (1.0)	0.450
Days sick before any health facility admission, median (IQR)	319	1 (0–7)	1 (0–4)	1 (0–7)	0.090
Days at district hospital prior to transfer, median (IQR)	243	1 (1–3)	2 (1–3)	1 (1–4)	0.927
**Hospital admission vital signs, median (IQR)**					
Temperature in °C	351	36.5 (36.0–37.2)	36.5 (36.0–37.0)	36.5 (36.0–37.7)	0.480
Systolic Blood Pressure in mmHg	419	124 (106–144)	125 (109–143)	123 (103–142)	0.306
Diastolic Blood Pressure in mmHg	419	73 (62–85)	74 (65–85)	72 (60–83)	0.183
Heart rate in beats per minute	417	104 (83–125)	102 (79–120)	109 (88–130)	0.007
Oxygen saturation	400	96 (91–98)	96 (93–99)	95 (90–98)	0.002
Receiving oxygen, n (%)	353	196 (55.5)	99 (55.0)	97 (56.7)	0.745
Respiratory Rate in breaths per minute	323	21 (18–27)	21 (18–26)	22 (20–28)	0.325
Glasgow Coma Scale	368	14 (9–15)	14 (10–15)	14 (8–15)	0.233

*n = number of patients. IQR = interquartile range*.

* Totals vary depending upon missing data for some patients.

**We could not locate in-hospital vital outcomes for two patients after extensive searching, so the number of patients in the survivor and non-survivor columns add to 425.

The most common reason for ICU admission was respiratory failure or need for endotracheal intubation (72.8%) (Table 2). Cardiopulmonary resuscitation (CPR) was performed within 24 hours before ICU admission for 7.5% (S2 Table). Within 24 hours of ICU admission, 42.2% had a diagnosis of sepsis, 33.0% severe sepsis, and 20.8% septic shock. ARDS criteria were met in 12.9% of all patients at any time during their ICU stay. In-hospital mortality was 48.7% (Table 3).

**Table 2:** Patient characteristics at ICU admission and during ICU stay.

	Number of Patients*	Full Cohort (n = 427)**	Survivors (n = 218)	Non-survivors (n = 207)	P Value
**ICU presenting characteristics, n (%)**					
ICU admission reason (non-exclusive categories)	427				
Respiratory failure		311 (72.8)	150 (68.8)	160 (77.3)	0.049
Altered mental status		138 (32.3)	56 (25.7)	82 (39.6)	0.002
Hypotension / shock		105 (24.6)	32 (14.7)	73 (35.3)	<0.001
Post-operative recovery		100 (23.4)	57 (26.2)	43 (20.8)	0.192
Sepsis		71 (16.6)	20 (9.2)	51 (24.6)	<0.001
Acute renal failure		45 (10.5)	22 (10.1)	23 (11.1)	0.733
Hemorrhage		34 (8.0)	19 (8.7)	15 (7.3)	0.577
Trauma		32 (7.5)	17 (7.8)	15 (7.3)	0.829
Seizure		29 (6.8)	14 (6.4)	14 (6.8)	0.887
Pre-eclampsia / eclampsia		13 (3.0)	9 (4.1)	4 (1.9)	0.189
Other		36 (8.4)	20 (9.2)	15 (7.3)	0.470
**Critical illness diagnoses, n (%)**					
Sepsis within 24 hours of ICU admission	427	180 (42.2)	63 (28.9)	116 (56.0)	<0.001
Severe sepsis within 24 hours of ICU admission	427	141 (33.0)	40 (18.4)	100 (48.3)	<0.001
Septic shock within 24 hours of ICU admission	427	89 (20.8)	15 (6.9)	74 (35.8)	<0.001
ARDS during ICU stay	427	55 (12.9)	13 (6.0)	41 (19.8)	<0.001
**ICU admission vital signs, median (IQR)**					
Temperature in °C	417	36.6 (36.0–37.5)	36.6 (36.0–37.2)	36.5 (35.8–37.9)	0.479
Systolic blood pressure in mmHg	422	115 (99–132)	117.0 (105–134)	113.0 (92–127)	0.003
Diastolic blood pressure in mmHg	422	71 (57–83)	74 (62–86)	69 (53–81)	<0.001
Heart rate in beats per minute	424	112 (96–130)	107 (90–120)	120 (105–138)	<0.001
Oxygen saturation	422	98 (94–100)	99 (95–100)	98 (93–100)	0.021
Respiratory rate in breaths per minute	387	20 (15–24)	20 (15–24)	20 (16–24)	0.356
Receiving oxygen, n (%)	425	411 (96.7)	208 (95.9)	201 (97.6)	0.323
Glasgow Coma Scale	272	8 (5–13)	10 (7–15)	6 (4–10)	<0.001

*n = number of patients. IQR = interquartile range*.

* Totals vary depending upon missing data for some patients.

**We could not locate in-hospital vital outcomes for two patients after extensive searching, so the number of patients in the survivor and non-survivor columns add to 425.

**Table 3:** ICU Interventions and Outcomes.

	Number of Patients*	Full Cohort (n = 427)**	Survivors (n = 218)	Non-survivors (n = 207)	P Value
**Interventions, n (%)**					
Surgery	427	296 (69.3)	154 (70.6)	141 (68.1)	0.572
Mechanical ventilation	427	364 (85.3)	163 (74.8)	199 (96.1)	<0.001
Days of mechanical ventilation, median (IQR)	359	2 (1–7)	2 (1–7)	2 (1–6)	0.868
Blood products	426	161 (37.8)	73 (33.5)	88 (42.7)	0.050
Vasopressors	425	178 (41.9)	40 (18.4)	137 (66.5)	<0.001
Renal replacement therapy	427	32 (7.5)	19 (8.7)	13 (6.3)	0.342
**Outcomes**					
ICU length of stay in days, median (IQR)	427	5 (3–9)	5 (3–9)	4 (2–8)	0.002
Hospital length of stay in days, median (IQR)	425	13 (6–27)	20 (12–41)	7 (4–14)	<0.001
In-hospital mortality, n (%)	425	207 (48.7)	0 (0)	207 (100)	<0.001

*n = number of patients. IQR = interquartile range*.

* Totals vary depending upon missing data for some patients.

**We could not locate in-hospital vital outcomes for two patients after extensive searching, so the number of patients in the survivor and non-survivor columns add to 425.

Surgical interventions were performed for 69.3% of all patients (Table 3). Mechanical ventilation was initiated for 85.3%, for a median of 2 (IQR 1–7) days. Blood products were given to 37.8%, and 41.9% received vasopressors. Renal replacement therapy was given to 7.5%. Median ICU length of stay was 5 (IQR 3–9) days for survivors and 4 (IQR 2–8) days for non-survivors. Median hospital length of stay was 20 (IQR 12–41) days for survivors and 7 (IQR 4–14) days for non-survivors.

### Rwanda-MPM model development

**Fig 1:**
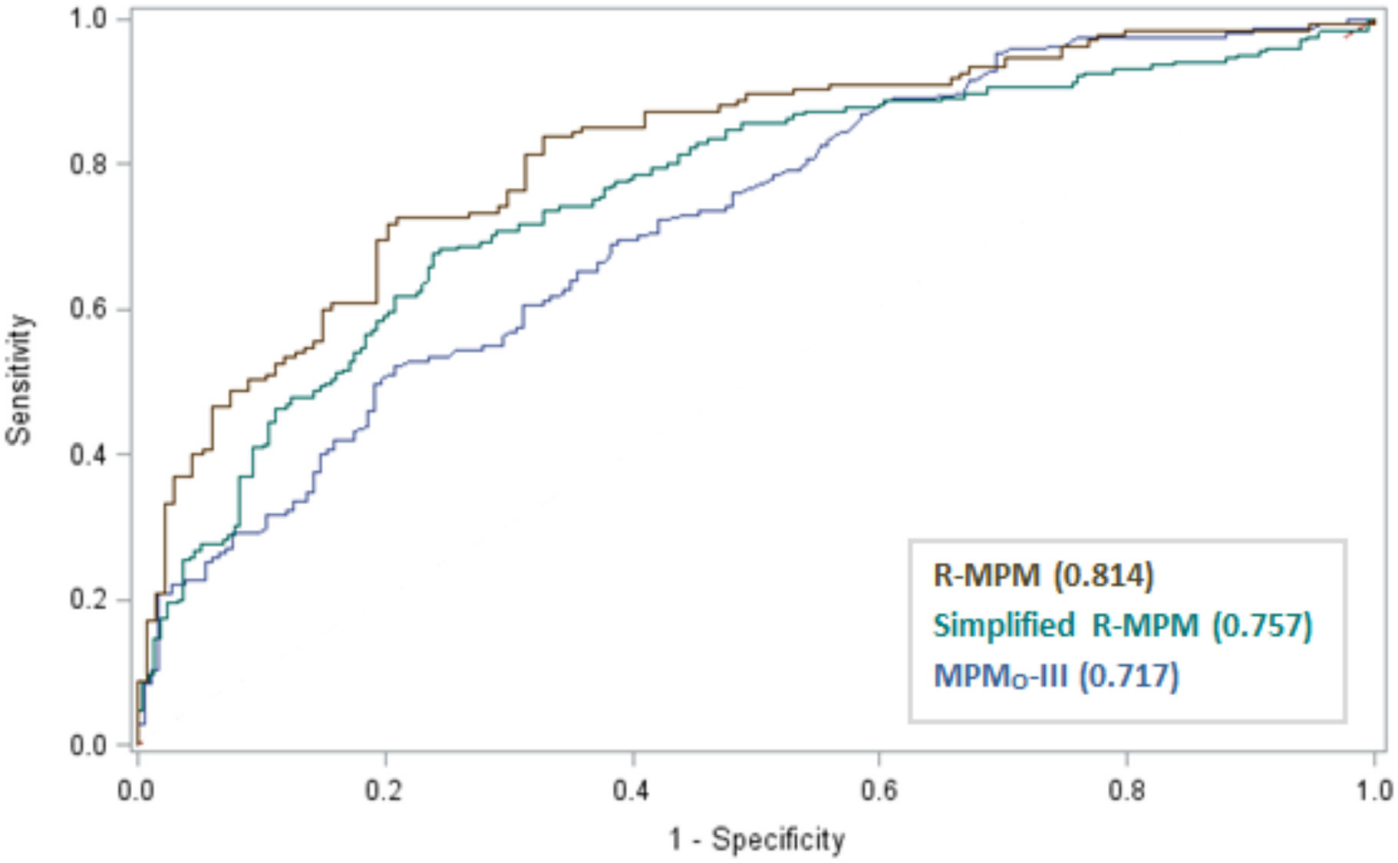
Receiver operating characteristic (ROC) curves for three risk prediction models in a Rwandan ICU population. *MPM_0_-III = Mortality Probability Admission Model, version III. R-MPM = Rwanda Mortality Probability Model as detailed in Table 4. Simplified R-MPM = the Rwanda Mortality Probability Model as detailed in Table 4 except that the variable altered mental status replaces the variable Glasgow Coma Scale score. The number in parentheses after each model name in the legend is the area under the ROC curve for that model*.

**Fig 2:**
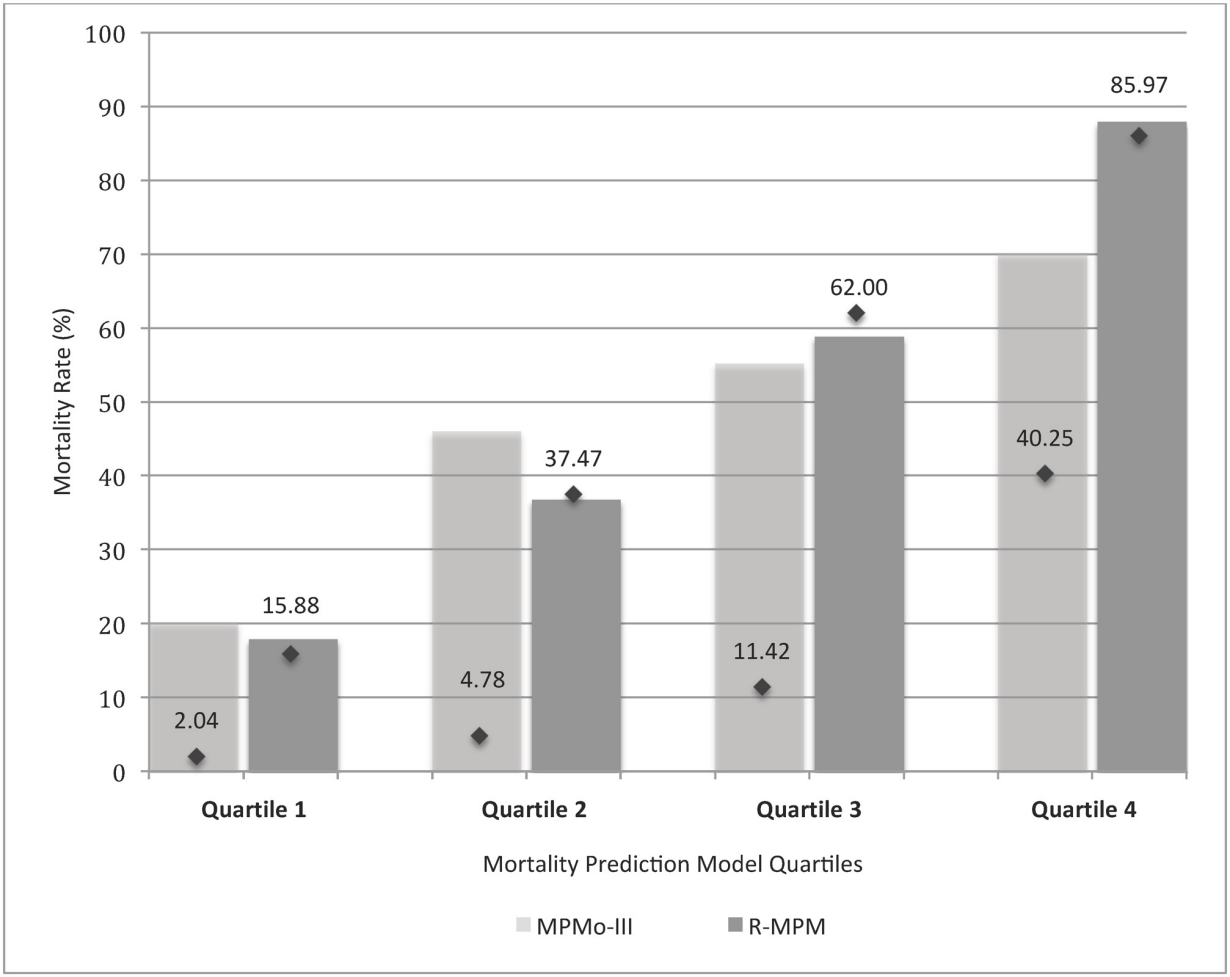
Expected and actual mortality rates by prediction model quartiles. *Each bar represents the actual mortality rate for that quartile, with quartiles determined by the specified risk prediction model. Each diamond represents the expected average mortality per quartile*.

The MPM_0_-III predicted mortality with area under the ROC curve of 0.72 (Hosmer-Lemeshow chi-square statistic of 17.66, p = 0.024.) (Table 4, Fig 1, and S4 Table) The variables that met our criteria for model inclusion were: age (OR 1.02, 95% CI 1.00–1.04, p = 0.019), confirmed or suspected infection within 24 hours of ICU admission (OR 3.14, 95% CI 1.74–5.70, p<0.001), hypotension or shock as a reason for ICU admission (see S1 Supporting Information for detailed description) (OR 2.54, 95% CI 1.20–5.42, p = 0.015), Glasgow Coma Scale (GCS) score at ICU admission (OR 0.79, 95% CI 0.74–0.86, p<0.0001), and heart rate at ICU admission (OR 1.02, 95% CI 1.01–1.03, p = 0.003) (Table 5). Using these five variables, the R-MPM predicted outcomes with area under the ROC curve of 0.81 (95% CI 0.77, 0.86) and Hosmer-Lemeshow chi-square statistic of 11.94, p = 0.154 (Table 4). Based on the odds ratios, the incremental change in mortality expected for a change in each independent variable is as follows: an increase in 10 years in age translates to an increase in the odds of death of 23%; patients with a suspected or confirmed infection within 24 hours of ICU admission have a 214% increase in the odds of death as compared to patients without; patients admitted to the ICU for shock or hypotension have a 155% increase in the odds of death as compared to those without shock or hypotension on admission; each increase in the GCS score by one point decreases the odds of death by 21%; and each 10-point increase in the heart rate translates to an increase in the odds of death of 19%. Fig 2 demonstrates the predicted versus actual mortality rates of the MPM_0_-III and R-MPM models by quartile.

**Table 4:** Discrimination and calibration of three risk prediction models in a Rwandan ICU population.

Risk prediction model	MPM_0_-III	R-MPM	Simplified R-MPM
AUC (0–1, higher values indicating better prediction)	0.72	0.81	0.76
Hosmer-Lemeshow chi-square statistic	17.66	11.94	11.46
Hosmer-Lemeshow p-value (higher p values indicating better model fit)	0.024	0.154	0.177
Brier score (0–1 scale, lower values indicating better prediction)	0.30	0.18	0.20
Adjusted R-Square (0–1, higher valued indicating better prediction)	0.16	0.37	0.25

*AUC = Area under the receiver operating characteristic curve. MPM_0_-III = Mortality Probability Admission Model, version III. R-MPM = Rwanda Mortality Probability Model. Simplified R-MPM = the Rwanda Mortality Probability Model except that the variable altered mental status replaces the variable Glasgow Coma Scale score*.

**Table 5:** Rwanda-Mortality Probability Model (R-MPM).

Parameter	OR (95% CI)	P Value
Age (per 10 years)	1.02 (1.00, 1.04)	0.019
Suspected or confirmed infection within 24 hours of ICU admission	3.14 (1.74, 5.70)	<0.001
Hypotension or shock as reason for ICU admission	2.54 (1.20, 5.42)	0.015
Glasgow Coma Scale (GCS) score at ICU Admission (per 1 point)	0.79 (0.74, 0.86)	<0.0001
Heart Rate at ICU Admission (per 10 points)	1.02 (1.01, 1.03)	0.003

While the GCS is documented on most patients in our dataset and in our clinical practice (63.7% have a score, 31.4% were sedated so had no numerical score, and 4.9% had a missing value in our dataset), we recognize that it may not be documented in all settings, and there is frequent uncertainty about scoring of verbal domains for endotracheally intubated and sedated patients. We therefore also examined a model using altered mental status on ICU admission (present versus not present) in place of the GCS score, the simplified R-MPM. This model gave an area under the ROC curve of 0.76 (Hosmer-Lemeshow chi-square statistic of 11.46, p = 0.177) (Table 4). Other than GCS, we had very few missing values for either the MPMo-III or R-MPM variables; all variables in the models had missing values <8% of the total (S3 Table). Just as in the original MPMo-III, a missing value was assumed to be normal for a given variable.

## Discussion

In this observational study of consecutive critically ill patients admitted to two ICUs in the low-income country of Rwanda we found that a series of five easily obtained bedside clinical variables have reasonable predictive ability, discrimination, and calibration. Our model performance compares favorably to other more complicated and less accessible clinical prediction systems (APACHE IV area under the ROC curve 0.88 and 0.86, Simplified Acute Physiology Score (SAPS3) 0.85 and 0.80, and Mortality Probability Admission Model (MPM_0_-III) 0.82 and 0.72 in a large validation cohort and single-center US study of 2596 patients, respectively.[1, 30])

The development of a risk prediction model in a low-income country is important because: 1) predictive models are essential for interpreting research and assessing quality of care for critically ill patients;[1] 2) models need to be calibrated to specific populations and contexts;[7, 20, 31] 3) models need to have reasonable data collection burden;[18] and 4) a model has not been developed or validated in a low-income country since 1989.[21]

While controversy exists about the value of risk prediction models for comparing ICUs to each other (ranking) in high-income countries, there is widespread agreement that the modeling of critical care outcomes is necessary for quality improvement efforts.[32] Even among high-income countries, the recalibration of models is deemed necessary to reflect differences in epidemiology and practices across countries.[7, 20, 31] It is not surprising that MPM_0_-III does not discriminate or calibrate well in our Rwandan ICU population. Even in gross comparisons, the Rwandan ICU population is markedly younger and more often has surgical diagnoses than the United States cohort on which MPM_0_-III was based. This is a common difference between low- and high-income settings, and is consistent with cohorts from other African ICUs.[5, 19] The issue of data collection burden, already prohibitive in many high-income countries[18], is even more salient in low-income countries, where ICUs do not have funding for data collection. The R-MPM model is more feasible for ongoing use due to its small number of variables and its focus on acute physiologic variables that can be easily assessed at the bedside. Finally, the last predictive model to be developed in a low-income country was Watters’ “Clinical Sickness Score” (CSS) in 1989 based on 624 ICU admissions to a university teaching hospital in Zambia, which yielded a sensitivity of only 36.4% and specificity of 93% in predicting survival. Contemporary statistical methods of developing and testing a model were not employed, and the model has not been updated to reflect changes in epidemiology or practice.[21]

Our model is simple to use and performs well in our development population with over one year of data collection to minimize seasonal bias. The model has several limitations. First, its relative simplicity will presumably translate to lower discrimination and calibration statistics when it is validated in a separate population. The tension between data collection burden and performance is not specific to low-income countries settings. One study applied the most common risk prediction models to 11,300 patients in 35 Californian ICUs and found excellent discrimination for all models (0.892, 0.873, 0.809 for APACHE IV, SAPS II, and MPM_0_-III respectively), but with time required to abstract the data in inverse relationship to model accuracy (37.3 minutes, 19.6 minutes, and 11.1 minutes, respectively).[33] Given the acceptable performance in all three models, using a model with lesser performance but lower data collection burden is thought to be a reasonable choice.[33]

Second, our model is based on a small sample size in two ICUs in a single country, and we were only able to perform internal validation using our development sample. The current validated models used cohorts ranging from 16,784 admissions for SAPS 3,[13] to 110,558 for APACHE IV,[4] to 124,855 for MPM_0_-III.[6] However, the first APACHE model was developed with only 805 admissions,[2] and the first MPM with 755.[34] Our model represents a foundation for ongoing work, which must include validation in future patients in the same ICUs as well as validation in other low-income-country ICUs.

Third, our model contains one variable, “suspected or confirmed infection within 24 hours of admission” that cannot be assessed at time of ICU admission. This is inconvenient in that it requires waiting 24 hours for full assessment. Its value may also be impacted by processes of care within the first 24 hours of ICU admission. While this is potentially problematic, ICU-acquired infections are generally not recognized within 24 hours, making this variable a marker of infections that began prior to ICU admission. Nonetheless, it may be worthwhile in future validations of the model to assess suspected or confirmed infection at the time of ICU admission.

Fourth, our model suffers from the same challenges as other risk prediction models: lead-time bias, the impact of pre-ICU and post-ICU care on outcomes, and the need for ongoing recalibration.[18] These are issues inherent to ICU risk prediction models, which can be partially mitigated by ongoing recalibration, but cannot be fully overcome. They are perhaps magnified in the mismatch of demand for and capacity of ICU beds, meaning that decision-making about who gets an ICU bed will have a large impact on the characteristics of ICU patients over time. Our model is based on patients admitted to two ICUs, a clinically-selected population that may not reflect the characteristics of all critically ill patients in the hospital. This is in fact one of the reasons that having a risk prediction model is so important in this setting—tracking outcomes over time may be impacted by changes in the ICU population over time, and a risk prediction model helps to control for such changes.

## Conclusions

Our study is the first performance-test of an ICU risk prediction model in a low-income setting and the first development of a new context-specific model in over twenty-five years. Our model is both better fit to our population and has a lower data collection burden than models developed in high-income countries. Our small sample size requires external validation in our own ICUs, other ICUs in low-income countries and in critically ill patients outside of ICUs. We are already aware of ICUs in three African countries that could apply this model with currently collected data. Just as large cohort databases have developed in the United States and Europe, so too a network of ICUs and hospitals from low-income countries could provide the necessary cohort to validate and reassess the model over time. While this presents a formidable challenge, we believe that both low- and high-income countries need ICU predictive models in order to effectively pursue quality improvement and research.[18] Our model is a starting place, and its choice of few and accessible variables means it can be readily assessed by other sites caring for critically ill patients in low-income countries.

## Supporting Information

S1 Supporting Information Reason for ICU admission. (DOCX)

S1 Table Additional patient characteristics at hospital admission. n = number of patients. IQR = interquartile range. HIV = human immunodeficiency virus. * Totals vary depending upon missing data for some patients. **We could not locate in-hospital vital outcomes for two patients after extensive searching, so the number of patients in the survivor and non-survivor columns add to 425.
(DOCX)

S2 Table Additional patient characteristics at ICU admission and during ICU stay. ICU = intensive care unit. n = number of patients. IQR = interquartile range. GCS = Glasgow Coma Scale. CPR = Cardiopulmonary resuscitation. * Totals vary depending upon missing data for some patients. **We could not locate in-hospital vital outcomes for two patients after extensive searching, so the number of patients in the survivor and non-survivor columns add to 425.
(DOCX)

S3 Table Missing values for variables in the MPMo-III and R-MPM models. GCS = Glasgow Coma Scale. CPR = Cardiopulmonary resuscitation. *The high proportion of missing values for GCS is driven by the many patients who had received sedating medications and could therefore not have GCS assessed. Of the 155 participants with missing GCS value, 134 were missing due to receiving sedating medications and 21 were missing due to having no GCS recorded.
(DOCX)

S4 Table Area under the ROC curve (C-statistic) for R-MPM model and for the model with each variable removed in turn. (DOCX)

## References

[1] Keegan MT, Gajic O, Afessa B. Severity of illness scoring systems in the intensive care unit. Crit Care Med. 2011;39(1):163–9. Epub 2010/09/15. . 20838329 10.1097/CCM.0b013e3181f96f81

[2] Knaus WA, Zimmerman JE, Wagner DP, Draper EA, Lawrence DE. APACHE-acute physiology and chronic health evaluation: a physiologically based classification system. Crit Care Med. 1981;9(8):591–7. Epub 1981/08/01. . 7261642 10.1097/00003246-198108000-00008

[3] Johnson AE, Kramer AA, Clifford GD. A new severity of illness scale using a subset of Acute Physiology And Chronic Health Evaluation data elements shows comparable predictive accuracy. Crit Care Med. 2013;41(7):1711–8. Epub 2013/05/11. . 23660729 10.1097/CCM.0b013e31828a24fe

[4] Zimmerman JE, Kramer AA, McNair DS, Malila FM. Acute Physiology and Chronic Health Evaluation (APACHE) IV: hospital mortality assessment for today's critically ill patients. Crit Care Med. 2006;34(5):1297–310. Epub 2006/03/17. . 16540951 10.1097/01.CCM.0000215112.84523.F0

[5] Higgins TL, Kramer AA, Nathanson BH, Copes W, Stark M, Teres D. Prospective validation of the intensive care unit admission Mortality Probability Model (MPM0-III). Crit Care Med. 2009;37(5):1619–23. Epub 2009/03/28. . 19325480 10.1097/CCM.0b013e31819ded31

[6] Higgins TL, Teres D, Copes WS, Nathanson BH, Stark M, Kramer AA. Assessing contemporary intensive care unit outcome: an updated Mortality Probability Admission Model (MPM0-III). Crit Care Med. 2007;35(3):827–35. Epub 2007/01/27. . 17255863 10.1097/01.CCM.0000257337.63529.9F

[7] Harrison DA, Brady AR, Parry GJ, Carpenter JR, Rowan K. Recalibration of risk prediction models in a large multicenter cohort of admissions to adult, general critical care units in the United Kingdom. Crit Care Med. 2006;34(5):1378–88. Epub 2006/03/25. . 16557153 10.1097/01.CCM.0000216702.94014.75

[8] Metnitz PG, Moreno RP, Almeida E, Jordan B, Bauer P, Campos RA, et al SAPS 3—From evaluation of the patient to evaluation of the intensive care unit. Part 1: Objectives, methods and cohort description. Intensive Care Med. 2005;31(10):1336–44. Epub 2005/09/01. 10.1007/s00134-005-2762-6 ; PubMed Central PMCID: PMCPmc1315314. 16132893 PMC1315314

[9] Kajdacsy-Balla Amaral AC, Andrade FM, Moreno R, Artigas A, Cantraine F, Vincent JL. Use of the sequential organ failure assessment score as a severity score. Intensive Care Med. 2005;31(2):243–9. Epub 2005/01/26. 10.1007/s00134-004-2528-6 . 15668764

[10] Marshall JC, Cook DJ, Christou NV, Bernard GR, Sprung CL, Sibbald WJ. Multiple organ dysfunction score: a reliable descriptor of a complex clinical outcome. Crit Care Med. 1995;23(10):1638–52. Epub 1995/10/01. . 7587228 10.1097/00003246-199510000-00007

[11] Cook R, Cook D, Tilley J, Lee K, Marshall J. Multiple organ dysfunction: baseline and serial component scores. Crit Care Med. 2001;29(11):2046–50. Epub 2001/11/09. . 11700393 10.1097/00003246-200111000-00002

[12] Harrison DA, Parry GJ, Carpenter JR, Short A, Rowan K. A new risk prediction model for critical care: the Intensive Care National Audit & Research Centre (ICNARC) model. Crit Care Med. 2007;35(4):1091–8. Epub 2007/03/06. . 17334248 10.1097/01.CCM.0000259468.24532.44

[13] Moreno RP, Metnitz PG, Almeida E, Jordan B, Bauer P, Campos RA, et al SAPS 3—From evaluation of the patient to evaluation of the intensive care unit. Part 2: Development of a prognostic model for hospital mortality at ICU admission. Intensive Care Med. 2005;31(10):1345–55. Epub 2005/09/01. 10.1007/s00134-005-2763-5 ; PubMed Central PMCID: PMCPmc1315315. 16132892 PMC1315315

[14] Paul E, Bailey M, Pilcher D. Risk prediction of hospital mortality for adult patients admitted to Australian and New Zealand intensive care units: development and validation of the Australian and New Zealand Risk of Death model. Journal of critical care. 2013;28(6):935–41. Epub 2013/10/01. 10.1016/j.jcrc.2013.07.058 . 24074958

[15] Adhikari NK, Fowler RA, Bhagwanjee S, Rubenfeld GD. Critical care and the global burden of critical illness in adults. Lancet. 2010;376(9749):1339–46. Epub 2010/10/12. S0140-6736(10)60446-1 [pii] 10.1016/S0140-6736(10)60446-1 . 20934212 PMC7136988

[16] Riviello ED, Letchford S, Achieng L, Newton MW. Critical care in resource-poor settings: lessons learned and future directions. Crit Care Med. 2011;39(4):860–7. Epub 2011/02/08. . 21297458 10.1097/CCM.0b013e318206d6d5

[17] The World Bank. Country and Lending Groups 2014 [cited 2014 July 8]. Available from: http://data.worldbank.org/about/country-and-lending-groups.

[18] Breslow MJ, Badawi O. Severity scoring in the critically ill: part 1—interpretation and accuracy of outcome prediction scoring systems. Chest. 2012;141(1):245–52. Epub 2012/01/05. 10.1378/chest.11-0330 . 22215834

[19] Kwizera A, Dunser M, Nakibuuka J. National intensive care unit bed capacity and ICU patient characteristics in a low income country. BMC Res Notes. 2012;5(1):475 Epub 2012/09/04. 1756-0500-5-475 [pii] 10.1186/1756-0500-5-475 . 22937769 PMC3470976

[20] Rivera-Fernandez R, Vazquez-Mata G, Bravo M, Aguayo-Hoyos E, Zimmerman J, Wagner D, et al The Apache III prognostic system: customized mortality predictions for Spanish ICU patients. Intensive Care Med. 1998;24(6):574–81. Epub 1998/07/29. . 9681779 10.1007/s001340050618

[21] Watters DA, Wilson IH, Sinclair JR, Ngandu N. A clinical sickness score for the critically ill in Central Africa. Intensive Care Med. 1989;15(7):467–70. Epub 1989/01/01. . 2600292 10.1007/BF00255604

[22] Annane D, Bellissant E, Cavaillon JM. Septic shock. Lancet. 2005;365(9453):63–78. Epub 2005/01/11. S0140-6736(04)17667-8 [pii] 10.1016/S0140-6736(04)17667-8 . 15639681

[23] Levy MM, Fink MP, Marshall JC, Abraham E, Angus D, Cook D, et al 2001 SCCM/ESICM/ACCP/ATS/SIS International Sepsis Definitions Conference. Crit Care Med. 2003;31(4):1250–6. Epub 2003/04/12. . 12682500 10.1097/01.CCM.0000050454.01978.3B

[24] Goldstein B, Giroir B, Randolph A. International pediatric sepsis consensus conference: definitions for sepsis and organ dysfunction in pediatrics. Pediatr Crit Care Med. 2005;6(1):2–8. Epub 2005/01/08. 01.PCC.0000149131.72248.E6 [pii] . 15636651 10.1097/01.PCC.0000149131.72248.E6

[25] Ranieri VM, Rubenfeld GD, Thompson BT, Ferguson ND, Caldwell E, Fan E, et al Acute respiratory distress syndrome: the Berlin Definition. JAMA. 2012;307(23):2526–33. Epub 2012/07/17. 1160659 [pii] 10.1001/jama.2012.5669 . 22797452

[26] Rice TW, Wheeler AP, Bernard GR, Hayden DL, Schoenfeld DA, Ware LB. Comparison of the SpO2/FIO2 ratio and the PaO2/FIO2 ratio in patients with acute lung injury or ARDS. Chest. 2007;132(2):410–7. Epub 2007/06/19. chest.07-0617 [pii] 10.1378/chest.07-0617 . 17573487

[27] Agency for Healthcare Research and Quality. Clinical Classifications Software (CCS) for ICD-9-CM 2014 [cited 2014 July 9]. Available from: http://www.hcup-us.ahrq.gov/toolssoftware/ccs/ccs.jsp.

[28] Harris PA, Taylor R, Thielke R, Payne J, Gonzalez N, Conde JG. Research electronic data capture (REDCap)—a metadata-driven methodology and workflow process for providing translational research informatics support. Journal of biomedical informatics. 2009;42(2):377–81. Epub 2008/10/22. ; PubMed Central PMCID: PMCPmc2700030. 18929686 10.1016/j.jbi.2008.08.010 PMC2700030

[29] Farmer PE, Nutt CT, Wagner CM, Sekabaraga C, Nuthulaganti T, Weigel JL, et al Reduced premature mortality in Rwanda: lessons from success. Bmj. 2013;346:f65 Epub 2013/01/22. 10.1136/bmj.f65 ; PubMed Central PMCID: PMCPmc3548616. 23335479 PMC3548616

[30] Keegan MT, Gajic O, Afessa B. Comparison of APACHE III, APACHE IV, SAPS 3, and MPM0III and influence of resuscitation status on model performance. Chest. 2012;142(4):851–8. Epub 2012/04/14. 10.1378/chest.11-2164 ; PubMed Central PMCID: PMCPmc3465106. 22499827 PMC3465106

[31] Vazquez Mata G, del Mar Jimenez Quintana M, Rivera Fernandez R, Bravo M, Aguayo De Hoyos E, Zimmerman J, et al [Severity assessment by APACHE III system in Spain]. Medicina clinica. 2001;117(12):446–51. Epub 2001/10/25. . 11674969 10.1016/s0025-7753(01)72141-0

[32] de Lange DW. The Pitfalls of Benchmarking ICUs*. Crit Care Med. 2015;43(2):473–4. Epub 2015/01/20. 10.1097/ccm.0000000000000732 . 25599469

[33] Kuzniewicz MW, Vasilevskis EE, Lane R, Dean ML, Trivedi NG, Rennie DJ, et al Variation in ICU risk-adjusted mortality: impact of methods of assessment and potential confounders. Chest. 2008;133(6):1319–27. Epub 2008/04/12. 10.1378/chest.07-3061 . 18403657

[34] Lemeshow S, Teres D, Pastides H, Avrunin JS, Steingrub JS. A method for predicting survival and mortality of ICU patients using objectively derived weights. Crit Care Med. 1985;13(7):519–25. Epub 1985/07/01. . 4006490 10.1097/00003246-198507000-00001

