# Supplementary material for: Predicting Mortality in Low-Income Country ICUs: The Rwanda Mortality Probability Model (R-MPM)
Source: PLoS One. 2016 May 19;11(5):e0155858. doi: 10.1371/journal.pone.0155858 (PMC4873171; doi:10.1371/journal.pone.0155858)
Supplement: S1 Supporting Information — (DOCX) [file pone.0155858.s001.docx]

**S1 Supplementary information. Reason for ICU admission.**

The variable “reason for ICU admission: hypotension or shock” is derived from a question on our case report form that allowed for multiple responses for ICU admission reason, including hypotension or shock. See below for the description from the operations manual used for training and reference for data collectors.

**Reason for ICU admission (choose *all* that apply):**

Include *all* reasons for ICU admission. This answers the question of why a patient needs ICU care. For example, if a patient comes for shock due to internal bleeding from a trauma after a motor vehicle accident, you would check shock, hemorrhage, trauma, and post-operative recovery.

🞏 Respiratory failure / respiratory distress / intubated

🞏 Hypotension / shock

🞏 Sepsis

🞏 Hemorrhage

🞏 Altered mental status

🞏 Acute renal failure

🞏 Trauma

🞏 Seizure

🞏 Pre-eclampsia / eclampsia

🞏 Post-operative recovery

🞏 Other, please specify ____________________
