# Supplementary material for: Predicting Mortality in Low-Income Country ICUs: The Rwanda Mortality Probability Model (R-MPM)
Source: PLoS One. 2016 May 19;11(5):e0155858. doi: 10.1371/journal.pone.0155858 (PMC4873171; doi:10.1371/journal.pone.0155858)
Supplement: S1 Table — n = number of patients. IQR = interquartile range. HIV = human immunodeficiency virus. * Totals vary depending upon missing data for some patients. **We could not locate in-hospital vital outcomes for two patients after extensive searching, so the number of patients in the survivor and non-survivor columns add to 425. (DOCX) [file pone.0155858.s002.docx]

**S1 Table. Additional patient characteristics at hospital admission.**

|  | | **Number of Patients*** | **Full Cohort**  **(n=427)**** | **Survivors**  **(n=218)** | **Non-survivors (n=207)** | **P Value** |
| --- | --- | --- | --- | --- | --- | --- |
| **Demographic characteristics, n (%)** | | | | | | |
| University Teaching Hospital of Kigali | | 427 | 234 (54.8) | 116 (53.2) | 116 (56.0) | 0.558 |
| **Hospital presenting characteristics, n (%)** | | | | | | |
| Hospital admission reason | Neurologic | 427 | 193 (45.2) | 99 (45.4) | 94 (45.4) | 0.999 |
|  | Surgical |  | 98 (23.0) | 56 (25.7) | 42 (20.3) | 0.187 |
|  | Gastrointestinal |  | 92 (21.6) | 32 (14.7) | 60 (29.0) | <0.001 |
|  | Obstetric Complications |  | 75 (17.6) | 49 (22.5) | 25 (12.1) | 0.005 |
|  | Infection |  | 55 (12.9) | 23 (10.6) | 32 (15.5) | 0.132 |
|  | Pulmonary |  | 53 (12.4) | 24 (11.0) | 29 (14.0) | 0.349 |
|  | Sepsis or Septic Shock |  | 37 (8.7) | 10 (4.6) | 27 (13.0) | 0.002 |
|  | Renal |  | 37 (8.7) | 25 (11.5) | 12 (5.8) | 0.038 |
|  | Cardiovascular |  | 32 (7.5) | 16 (7.3) | 16 (7.7) | 0.879 |
|  | Tumor |  | 29 (6.8) | 23 (10.6) | 6 (2.9) | 0.002 |
|  | Endocrine and Electrolyte Abnormalities |  | 12 (2.8) | 5 (2.3) | 6 (2.9) | 0.695 |
|  | Other |  | 30 (7.0) | 14 (6.4) | 16 (7.7) | 0.599 |
| Patient’s home in the same province as the hospital, n (%) | | 409 | 201 (49.1) | 95 (45.0) | 105 (53.6) | 0.085 |
| Hours travelled to first healthcare site, median (IQR) | | 297 | 0.5 (0–1) | 0.5 (0–1) | 0.5 (0–1) | 0.508 |
| HIV positive | | 427 | 23 (5.38) | 6 (2.90) | 16 (7.34) | 0.020 |

*n=number of patients. IQR = interquartile range. HIV = human immunodeficiency virus.*

** Totals vary depending upon missing data for some patients*

***We could not locate in-hospital vital outcomes for two patients after extensive searching, so the number of patients in the survivor and non-survivor columns add to 425.*
