# Supplementary material for: Predicting Mortality in Low-Income Country ICUs: The Rwanda Mortality Probability Model (R-MPM)
Source: PLoS One. 2016 May 19;11(5):e0155858. doi: 10.1371/journal.pone.0155858 (PMC4873171; doi:10.1371/journal.pone.0155858)
Supplement: S2 Table — ICU = intensive care unit. n = number of patients. IQR = interquartile range. GCS = Glasgow Coma Scale. CPR = Cardiopulmonary resuscitation. * Totals vary depending upon missing data for some patients. **We could not locate in-hospital vital outcomes for two patients after extensive searching, so the number of patients in the survivor and non-survivor columns add to 425. (DOCX) [file pone.0155858.s003.docx]

**S2 Table. Additional patient characteristics at ICU admission and during ICU stay.**

|  | | | **Number of Patients*** | **Full Cohort**  **(n=427)**** | **Survivors**  **(n=218)** | **Non-survivors (n=207)** | **P Value** |
| --- | --- | --- | --- | --- | --- | --- | --- |
| **ICU presenting characteristics, n (%)** | | | | | | | |
| Location prior to ICU admission | Operating theatre | | 427 | 153 (35.8) | 89 (40.8) | 64 (30.9) | 0.033 |
|  | Emergency room | |  | 70 (16.4) | 34 (15.6) | 35 (16.9) | 0.714 |
|  | Post-operative recovery room | |  | 62 (14.5) | 26 (11.9) | 36 (17.4) | 0.111 |
|  | Medical ward | |  | 56 (13.1) | 25 (11.5) | 31 (15.0) | 0.285 |
|  | Obstetrics and gynecology ward | |  | 48 (11.2) | 25 (11.5) | 22 (10.6) | 0.783 |
|  | Obstetric recovery room | |  | 21 (4.9) | 7 (3.2) | 14 (6.8) | 0.091 |
|  | Direct transfer from another hospital | |  | 7 (1.6) | 6 (2.8) | 1 (0.5) | 0.066 |
|  | Surgical ward | |  | 5 (1.2) | 3 (1.4) | 2 (1.0) | 0.695 |
|  | Other | |  | 5 (1.12) | 3 (1.4) | 2 (1.0) | 0.695 |
| **Lab values within 24 hours of ICU admission, median (IQR)** | | | | | | | |
| Sodium (mEq/L) | | | 288 | 139 (135-143) | 139 (135-142) | 139 (136-145) | 0.076 |
| Potassium (mEq/L) | | | 287 | 4.2 (3.7-4.9) | 4.1 (3.7-4.5) | 4.5 (3.7-5.4) | 0.003 |
| Chloride (mEq/L) | | | 246 | 105 (100-110) | 103 (100-108) | 106 (101-114) | 0.001 |
| Blood urea (mg/dL) | | | 214 | 8 (4-23) | 67 (4-17) | 11 (5-28) | 0.038 |
| Hemoglobin (g/dL) | | | 367 | 11.4 (9.4-13.8) | 11.6 (9.5-14.1) | 11.3 (8.9-13.5) | 0.252 |
| White blood cells (1,000s/mm^3^) | | | 352 | 11.9 (7.9-16.9) | 11.4 (7.9-16.2) | 12.1 (7.9-17.3) | 0.703 |
| Platelets (mg/dL) | | | 354 | 164.5 (95.0-256.0) | 183.0 (109.0-274.0) | 145.0 (84.0-231.0) | 0.007 |
| Aspartate transaminase (AST) (U/L) | | | 201 | 61 (32-112) | 51 (28-90) | 76 (42-197) | 0.003 |
| Alanine transaminase (ALT) (U/L) | | | 222 | 34 (20-71) | 33 (17-62) | 37 (23-75) | 0.070 |
| **MPM_0_-III** **characteristics** | | | | | | | |
| Hospital Admission Type | | Unscheduled Surgical | 427 | 198 (46.4) | 95 (43.6) | 103 (49.8) | 0.202 |
|  |  | Medical |  | 164 (38.4) | 79 (36.2) | 83 (40.1) | 0.413 |
|  |  | Scheduled Surgical |  | 65 (15.2) | 44 (20.2) | 21 (10.1) | 0.004 |
| “Full code” status on ICU admission | | | 426 | 422 (99.1) | 215 (99.1) | 205 (99.0) | 0.962 |
| CPR within 24 hours prior to ICU admission | | | 425 | 32 (7.5) | 8 (3.7) | 22 (10.7) | 0.005 |
| Coma (GCS 3-5) on ICU admission | | | 272 | 80 (29.4) | 18 (13.3) | 61 (44.9) | <0.001 |
| Heart rate ≥ 150 beats per minute on ICU admission | | | 424 | 25 (5.9) | 3 (1.4) | 22 (10.7) | <0.001 |
| Systolic blood pressure ≤ 90 on ICU admission | | | 422 | 69 (16.4) | 23 (10.6) | 46 (22.7) | <0.001 |
| Mechanical ventilation within one hour of ICU admission | | | 426 | 341 (80.1) | 151 (69.6) | 188 (90.8) | <0.001 |
| Acute renal failure | | | 408 | 108 (26.5) | 41 (19.3) | 66 (34.0) | <0.001 |
| Cardiac dysrhythmias | | | 424 | 38 (9.0) | 13 (6.1) | 24 (11.6) | 0.044 |
| Cerebrovascular accident | | | 414 | 19 (4.6) | 10 (4.8) | 9 (4.4) | 0.865 |
| Intracranial mass effect | | | 411 | 31 (7.5) | 15 (7.1) | 16 (8.0) | 0.732 |
| Gastrointestinal bleeding | | | 417 | 17 (4.1) | 3 (1.4) | 14 (7.0) | 0.004 |
| Metastatic carcinoma | | | 426 | 9 (2.1) | 4 (1.8) | 5 (2.4) | 0.745 |
| Chronic renal insufficiency | | | 393 | 41 (10.4) | 13 (6.4) | 27 (14.4) | 0.010 |
| Cirrhosis | | | 425 | 6 (1.4) | 3 (1.4) | 3 (1.5) | 0.940 |

*ICU = intensive care unit. n=number of patients. IQR = interquartile range. GCS = Glasgow Coma Scale. CPR = Cardiopulmonary resuscitation*.

** Totals vary depending upon missing data for some patients*

***We could not locate in-hospital vital outcomes for two patients after extensive searching, so the number of patients in the survivor and non-survivor columns add to 425.*
