# Supplementary material for: Predicting Mortality in Low-Income Country ICUs: The Rwanda Mortality Probability Model (R-MPM)
Source: PLoS One. 2016 May 19;11(5):e0155858. doi: 10.1371/journal.pone.0155858 (PMC4873171; doi:10.1371/journal.pone.0155858)
Supplement: S3 Table — GCS = Glasgow Coma Scale. CPR = Cardiopulmonary resuscitation. *The high proportion of missing values for GCS is driven by the many patients who had received sedating medications and could therefore not have GCS assessed. Of the 155 participants with missing GCS value, 134 were missing due to receiving sedating medications and 21 were missing due to having no GCS recorded. (DOCX) [file pone.0155858.s004.docx]

**S3 Table. Missing values for variables in the MPMo-III and R-MPM models.**

| **Total participants, n=427** | **Missing Values** | **% Missing Values** |
| --- | --- | --- |
| **MPM Variables** | | |
| GCS (Coma)* | 155 | 36.30 |
| Heart Rate (HR > 150) | 3 | 0.70 |
| Systolic Blood Pressure (SBP < 90) | 5 | 1.17 |
| Chronic renal compromise / Insufficiency | 34 | 7.96 |
| Cirrhosis | 2 | 0.47 |
| Metastatic Malignant neoplasm | 1 | 0.23 |
| Acute Renal Failure | 19 | 4.45 |
| Cardiac Dysrhythmia | 3 | 0.70 |
| Cerebrovascular Incident | 13 | 3.04 |
| Gastrointestinal Bleeding | 10 | 2.34 |
| Intracranial Mass Effect | 16 | 3.75 |
| CPR | 2 | 0.47 |
| Mechanical Ventilation | 1 | 0.23 |
| Medical / Unscheduled Surgery Admission | 0 | 0.00 |
| Full Code Status | 1 | 0.23 |
| Age | 0 | 0.00 |
| **R-MPM / Simplified R-MPM Variables** | | |
| Age | 0 | 0.00 |
| GCS* | 155 | 36.30 |
| Suspected / confirmed infection | 0 | 0.00 |
| Hypotension / shock | 0 | 0.00 |
| Heart Rate | 3 | 0.70 |
| AMS at admission | 0 | 0.00 |

*GCS = Glasgow Coma Scale. CPR = Cardiopulmonary resuscitation*.

**The high proportion of missing values for GCS is driven by the many patients who had received sedating medications and could therefore not have GCS assessed. Of the 155 participants with missing GCS value, 134 were missing due to receiving sedating medications and 21 were missing due to having no GCS recorded.*
