# Supplementary material for: Predicting Mortality in Low-Income Country ICUs: The Rwanda Mortality Probability Model (R-MPM)
Source: PLoS One. 2016 May 19;11(5):e0155858. doi: 10.1371/journal.pone.0155858 (PMC4873171; doi:10.1371/journal.pone.0155858)
Supplement: S4 Table — (DOCX) [file pone.0155858.s005.docx]

**S4 Table. Area under the ROC curve (C-statistic) for R-MPM model and for the model with each variable removed in turn.**

|  | **C Statistic** | **Hosmer-Lemeshow p value** |
| --- | --- | --- |
| **R-MPM** | 0.814 | 0.154 |
| **R-MPM Model without the variable:** |  |  |
| Age | 0.801 | 0.531 |
| Suspected / confirmed infection within 24 hours of ICU admission | 0.786 | 0.204 |
| Hypotension or shock as reason for ICU admission | 0.802 | 0.969 |
| Glasgow Coma Scale (GCS) score at ICU Admission | 0.742 | 0.228 |
| Heart Rate at ICU Admission | 0.799 | 0.966 |
